# Supplementary material for: Home delivery practice and its predictors in South Ethiopia
Source: PLoS One. 2021 Aug 9;16(8):e0254696. doi: 10.1371/journal.pone.0254696 (PMC8351986; doi:10.1371/journal.pone.0254696)
Supplement: S1 Questionnaire — (DOC) [file pone.0254696.s003.doc]

**Hawassa University, College of Medicine and Health Sciences**

**A questionnaire designed to collect data on place of delivery and child care practice in Hawassa City, Dale and Shebedino Districts.**

**Instruction:** Please, read each question to the respondent slowly and write the responses on the blank spaces or circle the respondent’s choice.

| **Woreda’s name __________________** | | | **Code_________________** | | |
| --- | --- | --- | --- | --- | --- |
| **KEBELE’s Name____________________** | | | **Code ________________** | | |
| **HOUSEHOLD NUMBER (If any)** | | | **[_______|________|_______]** | | |
| **Child’s Birth Date** | | | ________/__________/______________,  Day Month Year | | |
| **(Child‘s age), If deceased, record age at death.** | | | **In Weeks** [_____ | **_____] or Months** --------------- | | |
| **Child (NAME) sex** | | | 1…..Male  2…...Female | | |
| **Section 1. Respondent and Household Characteristics** | | | | | |
| S.No | Variables | Categories | | | SKIP |
| 101 | How old are you? | Age in completed years [_____ | ______] | | |  |
| 102 | What is the highest class you completed | No class attended………………….……0  Grade 1 to 4…………………….…….. .1  Grade 5 to 8…………………………….2  Grade 9 to 10 …………………………..3  Higher than Grade 10 ………………… 4 | | |  |
| 103 | What is your occupation? | Farmer ...................................................1  Housewife ..............................................2  Daily laborer………...............................3  Merchants/trader………... .....................4  Employee (government/private business/NGO)….…...............................5  Other *(specify)* ........................................6 | | |  |
| 104 | What is the highest level of education your spouse attended and completed? | Not attended formal school……………1  Incomplete Primary………..……..........2  Complete Primary………........................3  Any Secondary or Higher………….......4 | | |  |
| 105 | What is your household’s average monthly income? | income: ___________Birr  Do not know…………………………..99 | | |  |
| 106 | How long does it take you to walk to the nearest health center?  What about the health post?  ***If less than an hour, record it in minutes, and write 00 in space for hour.*** | | | HEALTH CENTER  Minutes [____|____] Hours [____|____]  HEALTH POST  Minutes [____|____] Hours [____|____] |  |
| 107 | Total number of the family members in this household? | | | _________ |  |
| 108 | In your life time, how many times have you been pregnant?  Total life births?  Abortions?  Still birth/IUFD?  Neonatal death | | | Pregnancy, __________________  Life birth, __________________  Abortions, __________________  Still birth/IUFD, _____________  Neonatal Death ______________ |  |
| 109 | What is birth order of the last neonate? | | | __________________________ |  |

| **Section 2. Antenatal Care and birth preparedness plan**  *Now I would like to ask you some questions about services you may have received during your pregnancy* | | | | |
| --- | --- | --- | --- | --- |
| 201 | Did you have ANC follow up for your index child? | Yes………………………………….1  No………………………………….2 |  | |
| 202 | Where did you receive antenatal care for this pregnancy? Anywhere else?  ***Probe to identify type(s) of source(s) and circle the appropriate code(s). Circle all mentioned.*** | Mentioned: Yes No   1. Govt. Hospital. . . . .......1 2 2. Govt. Health center ..... 1 2 3. Govt. Health post…… .1 2 4. NGO Clinic………… ...1 2 5. Other Specify___________________ |  | |
| 203 | How many times did you receive antenatal care during pregnancy for this infant? | Number of times _______________________ |  | |
| 204 | During your last pregnancy, did you know your due date, or when you would expect to deliver the baby? | Yes…………………………….1  No……………………………..2 |  | |
| 205 | Whom did you plan to attend your delivery?  ***DO NOT READ OUT ANSWERS****.*  ***Probe: “Did you do plan anyone else to attend the delivery?”***  **(Circle all responses)** | Mentioned: Yes No   1. Health worker at facility …..1 2 2. Traditional Birth Attendant..1 2 3. Mother……………………...1 2 4. Mother-in-law………………1 2 5. Other female relative…….….1 2 6. Health Extension Worker…...1 2 7. Community health volunteer..1 2 8. Other specify __________________ 9. No one………………………...1 2 |  | |
| 206 | During your last delivery did you plan for a place to deliver your child? | Yes……………………………….…….1  No………………………………………2 |  | |
| 207 | Where did you plan to deliver your last child? | HOME  Your home/other home. .……….. 1  PUBLIC SECTOR  Govt. Hospital…………………….2  Govt. Health center . . . . . ……. …..3  Govt. clinic………………………….4  NON-GOVT(NGO) HEALTH FACILITY…5  PRIVATE MED. SECTOR  Pvt. Hospital/clinic.……………..…. 6  OTHER specify__________________­­­_____ |  | |
| **Section 3. Delivery and Immediate Newborn Care** | | | | |
| 301 | Mode of delivery of the current (index) child | SVD………………………..….1  C/S……………………………2  Instrumental…………………..3 |  | |
| 302 | Who assisted with the delivery of (NAME)?  Anyone else?  Probe for the type(s) of person(s) and record all mentioned. If respondent says ‘no one assisted’, probe to determine whether any adults were present at the delivery. | Mentioned: Yes No  HEALTH PERSONNEL   1. Doctor ………………….....1 2 2. Nurse/midwife.…. …….….. 1 2 3. Health extension worker……1 2 4. Health worker unknown type.1 2   OTHER PERSON   1. Traditional birth attendant. .1 2 2. Relative/friend…………..…1 2 3. Other specify_____________________   No One . . . . …………………………….…..1 2 |  | |
| 303 | Where was the baby placed immediately after delivery? | On the floor …………….………….… 1  On the mother’s belly/chest..……..….. 2  Beside the mother……………………...3  With someone else …………………... 4  On newborn bed/table………………...5  Other (specify)__________________________.  Don’t know …………………………….... 9 | |  |
| 304 | Who took care of the newborn? | HEALTH PERSONNEL  Nurse/midwife…. …………………… 1  Health extension worker………………2  Health worker unknown type…………3  OTHER PERSONS  Traditional birth attendant. ……….….4  Mother/Mother-in-law………………..5  Sister/sister-in-law…………………….6  Other female relative………………….7  Other specify________________________  NO ONE ... . ……………………………......... 9 | |  |
| 305 | What was used to tie the cord? | New string or thread……………………….....1  String, or thread …………….………..............2  Cord was not tied …………………………. .3  Other specify …………………………… 4  Don’t know …………………………...9 | |  |
| 306 | What was used to cut the cord? | New razor blade…............................................1  Razor blade …………………………………..2  Scissors……………………………………….3  Other specify.....................................................4  Don’t Know/Can’t Remember…......................9 | |  |
| 307 | What was applied to the cord just after cutting the cord? | Butter…………………………….…………...…1  Ash………………………………………………2  Ointment……….……………..…………………3  Animal dung…………………...………….........4  Oil….……………………………………..…….5  Cold water…………………………….………..6  Other………………………………………......7  Other (specify) _______________________  Don’t know…………………….………………9 | |  |
| 308 | How much did (NAME) weigh at birth?  ***RECORD WEIGHT FROM HEALTH***  ***CARD, IF AVAILABLE.*** | KG from recall______.______  Baby not weighed…………..………..96  Don’t know………. ……………..…..98 | |  |
| 309 | How often was (name) breastfed or cup fed?  Probe for number of times fed during the day and number of times fed during the night | ______________number of times | |  |
| 310 | Where did you give birth to (NAME)?  Probe to identify the type of source and circle the appropriate code.  If unable to determine if a hospital, health center, or clinic is public or private medical, write the name of the place.  ____________________________________  (NAME OF PLACE) | **HOME**  Your home/other home. . ………….……… 1  **PUBLIC SECTOR**  Govt. Hospital……………………..……….2  Govt. Health center . . . . . …………... …….3  Govt. clinic………………………………….4  Govt. Health post . . . . . . . ………..………. 5  NON-GOVT(NGO) HEALTH FACILITY …6  PRIVATE MED. SECTOR ………………....7  Pvt. Hospital/clinic.………………….……..…. 8  OTHER specify__________________­­­­_____________ | |  |
| 311 | What was the reason you didn’t deliver in a health facility?  Do Not Read Out Responses  PROBE: Any other reason?  (Record all mentioned). | Mentioned: Yes No   1. Preferred to deliver at home……….. .1 2 2. Cost too much. ……………… ………1 2 3. Too far/ no transportation…………….1 2 4. Not necessary …………………………1 2 5. Not customary…………………………1 2 6. Delivered on the way to health facility………………………….............1 2 7. Other specify ……………………………… | |  |
