# Supplementary material for: Home delivery practice and its predictors in South Ethiopia
Source: PLoS One. 2021 Aug 9;16(8):e0254696. doi: 10.1371/journal.pone.0254696 (PMC8351986; doi:10.1371/journal.pone.0254696)
Supplement: Attachment — Submitted filename: Point by point response.docx [file pone.0254696.s004.docx]

Dear Dr Kassa,

Thank you for submitting your manuscript entitled "Home Delivery Practice and its Predictors in South Ethiopia" to PLOS ONE.  Your manuscript files have been checked in-house but before we can proceed we need you to address the following issues:

1.  Thank you for updating your manuscript according to our requests. I apologize that we are requesting further updates, but it is PLOS ONE's goal to provide the highest standard of reporting in manuscripts that are published with us and there are a few more points we would like to clarify before we can proceed. Please update your ethics statement in the Methods and the online submission form to include the following information:

* Was the ethics committee aware that oral consent was collected rather than written consent. Did the ethics committee specifically approved this consenting procedure?

- **Response:** Thank you for the comment. Yes, the Institutional Review Board (IRB) of Hawassa University is aware that this was an oral consent rather than written and approved this consenting procedure..

* Did the ethics committee approve your study protocol and consent procedure knowing that some of the participants were <18 years of age? Did they require any form of parental consent?

- **Response:** The IRB is well aware that the respondents participated in this survey were married and already left their parents. If a woman is married at age less than 18 years old, then, she leaves her parents and lead her life independent of her parents. The IRB knows that this a reality in Ethiopia and thus, approved the consent without requiring further parental consent.

Your manuscript has been returned to your account. Please log on to PLOS Editorial Manager at <https://www.editorialmanager.com/pone/> to access your manuscript.

Your manuscript can be found in the "Revisions Sent Back to the Author" link under the New Submissions menu. After you have made the changes requested above, please be sure to view and approve the revised PDF after rebuilding the PDF to complete the resubmission process.

Please note that these changes have been requested to comply with submission guidelines and your manuscript will *not* be sent to review until you have fully adhered to our requests.  Once your paper has been seen by an Editor we may return it to you for further information or amendments.

We ask that you address this request within 21 days. If you require additional time, please email the journal office. We are happy to grant extensions of up to one month past this due date. If we have not heard from you within 21 days, your manuscript will be withdrawn from Editorial Manager.

Thank you for submitting your work to PLOS ONE.

Kind regards,

Jo George

PLOS ONE
